# Use of chlorhexidine-impregnated dressing in neonates

**DOI:** 10.1186/1753-6561-5-S6-P61

**Published:** 2011-06-29

**Authors:** R Richtmann, C Silva, S Baltieri, T Rodrigues, F Camolesi, E Quadrado, K Saito, C Andrade

**Affiliations:** 1 Hospital e Maternidade Santa Joana JOANA, São Paulo, Brazil

## Introduction

Catheter–related bloodstream infection (CR-BSI) is a significant cause of morbidity and mortality in the NICU. The chlorhexidine-impregnated dressing (CHID) has proven effective in reducing the colonization catheter tip and CR-BSI.

## Objectives

Describe the experience on using CHID (Biopatch®) on the central venous catheter (CVC) in neonates at a 70 beds NICU.

## Methods

The NICU has a group responsible for the insertion and maintenance of CVC according to the standards recommendation from CDC.InMay2010we startedusing the CHID in all neonates weighing > 1500g and gestational age > 34 weeks. The dressing was replaced weekly. The observation was conducted from May to December 2010. Besides the new dressing, we also changed from 70% alcohol to alcoholic chlorhexidine solution for disinfection of the CVC hub.

## Results

**Sixty-four** neonates were enrolled in the trial**.** There was no CR-BSI in neonates weighing > 1500g after the introduction of the CHID (0 / 1089 cvc /day). Compared to the same period in 2009 (without Biopatch), the rate of CR-BSI was 3.2 (6 / 1833 cvc / day). One neonate developed localized contact dermatitis with absolute regression after removal of the dressing.

## Conclusion

We are not able to affirm that this intervention was responsible for the decrease of CR_BSI rate, but these dataisencouraging. Local contact dermatitis was not a problem. There was a good acceptance of the new dressing by the nursing staff. Apparently the CHID is safe and significantly reduces the rates of CR-BSI in neonates.

## Disclosure of interest

None declared.

